# Supplementary material for: DMRforPairs: identifying Differentially Methylated Regions between unique samples using array based methylation profiles
Source: BMC Bioinformatics. 2014 May 15;15:141. doi: 10.1186/1471-2105-15-141 (PMC4046028; doi:10.1186/1471-2105-15-141)
Supplement: Additional file 2 — DMRforPairs output for the comparison of A431-MCF7 and NA17018-NA17105. Please start from the HTML files in each folder. Available via the BMC Bioinformatics website. [file 1471-2105-15-141-S2.zip › 1394847754114233_MOESM2_ESM/A431_MCF7/figures/12649.pdf]

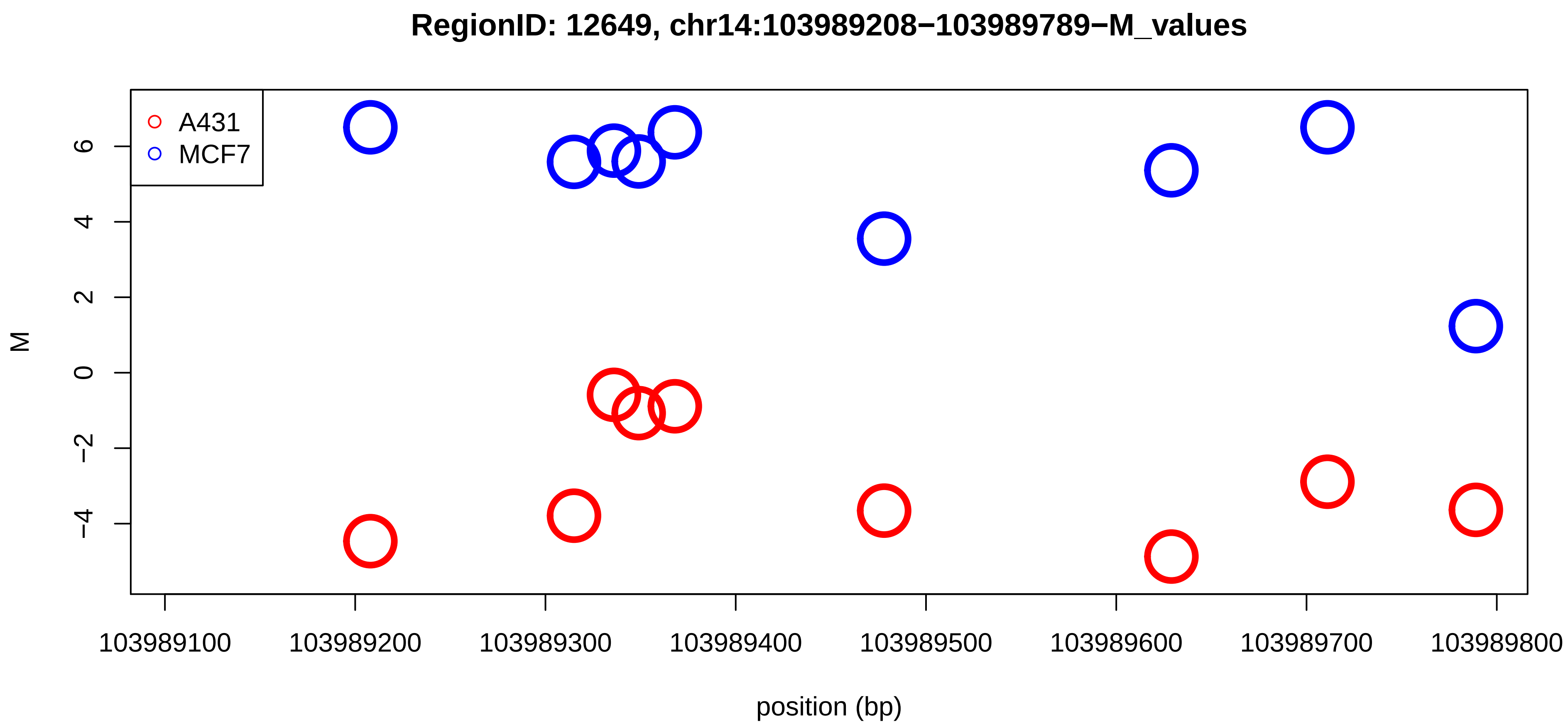
RegionID: 12649, chr14:103989208−103989789−M_values
A431
MCF7
M
6
4
2
0
−2
−4
103989100
103989200
103989300
103989400
103989500
103989600
103989700
103989800
position (bp)

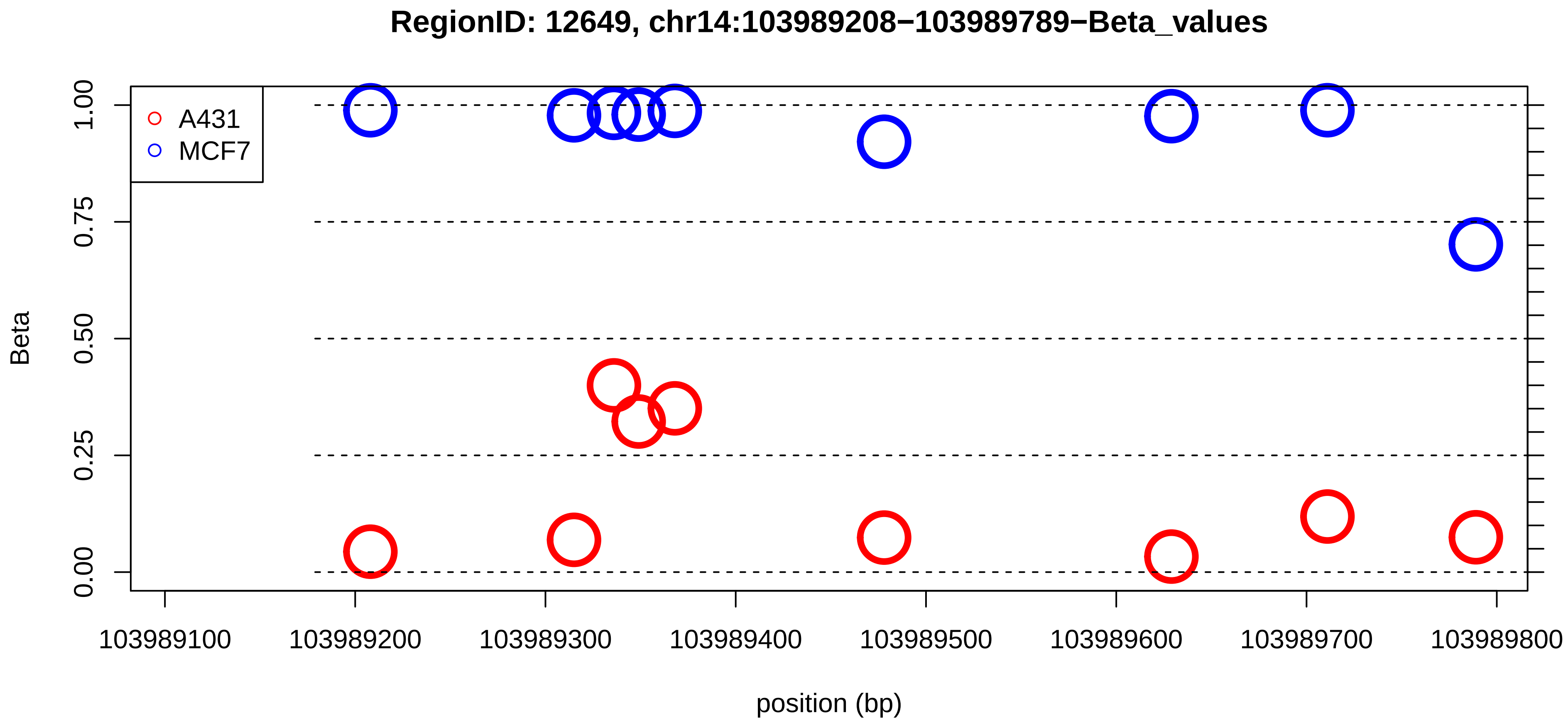
RegionID: 12649, chr14:103989208−103989789−Beta_values
A431
MCF7
Beta
1.00
0.75
0.50
0.25
0.00
103989100
103989200
103989300
103989400
103989500
103989600
103989700
103989800
position (bp)

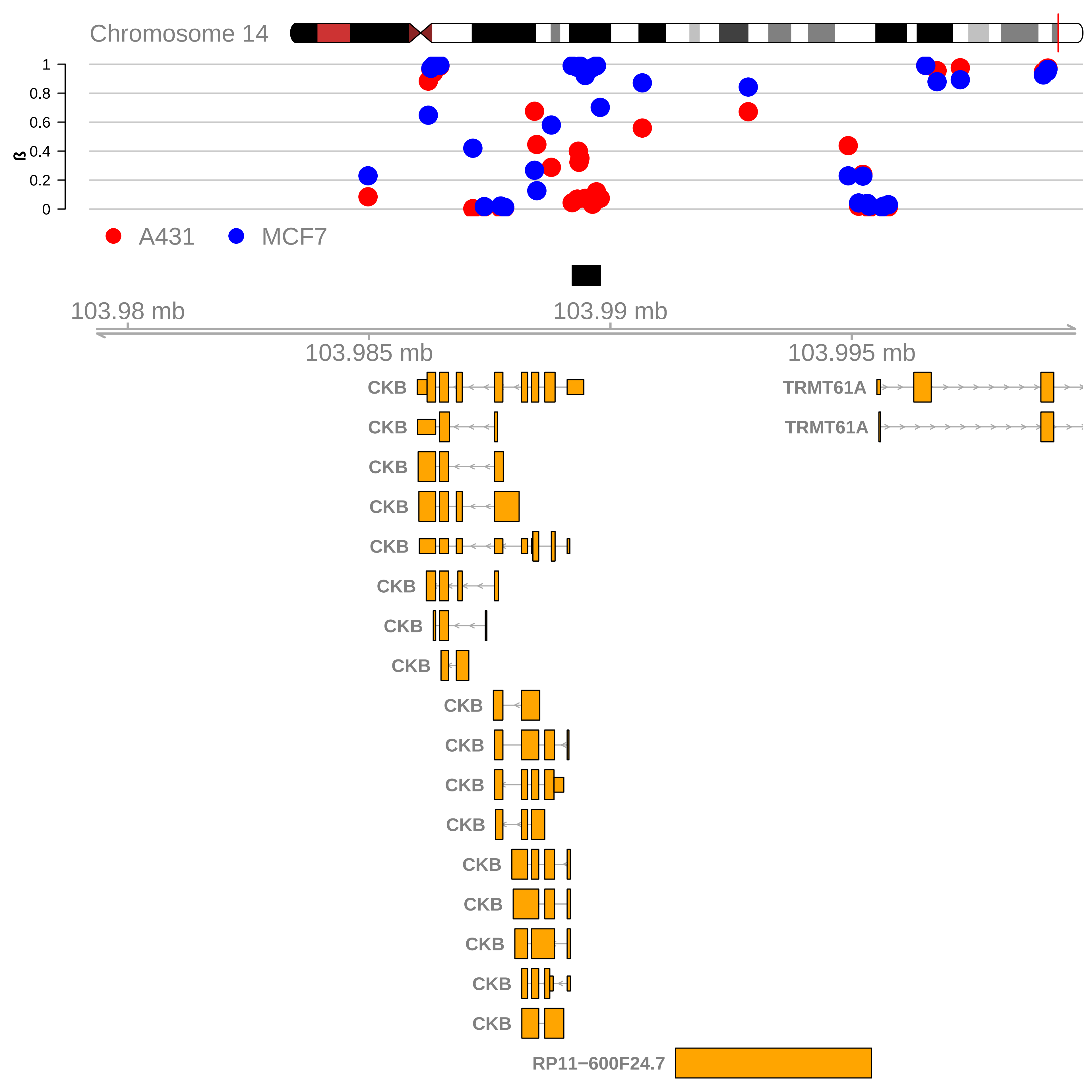

Chromosome 14
1
0.8
0.6
0.4
0.2
0
ß
A431
MCF7
103.98 mb
103.99 mb
103.985 mb
103.995 mb
CKB
TRMT61A
RP11−600F24.7
